# Prevalence of cystic echinococcosis and associated risk factors among humans in Khartoum State, Central Sudan

**DOI:** 10.1093/inthealth/ihaa059

**Published:** 2020-09-19

**Authors:** Mohamed E Ahmed, Sara Siddig Abdalla, Ibrahim A Adam, Martin P Grobusch, Imadeldin E Aradaib

**Affiliations:** Echinococcosis Research Center, Zamzam University College, Khartoum, Sudan; Department of Surgery, Faculty of Medicine, Al-Neelain University, Khartoum, Sudan; Molecular Biology Laboratory, Faculty of Veterinary Medicine, University of Khartoum, Sudan; Echinococcosis Research Center, Zamzam University College, Khartoum, Sudan; Molecular Biology Laboratory, Faculty of Veterinary Medicine, University of Khartoum, Sudan; Faculty of Medical Laboratory, Al-Neelain University, Khartoum, Sudan; Molecular Biology Laboratory, Faculty of Veterinary Medicine, University of Khartoum, Sudan; Center of Tropical Medicine and Travel Medicine, Department of Infectious Diseases, Amsterdam Public Health, Amsterdam Infection and Immunity, Amsterdam University Medical Centers, University of Amsterdam, Amsterdam, The Netherlands; Echinococcosis Research Center, Zamzam University College, Khartoum, Sudan; Molecular Biology Laboratory, Faculty of Veterinary Medicine, University of Khartoum, Sudan

**Keywords:** *Echinococcus granulosus*, ELISA, epidemiology, hydatid disease, Sudan, survey

## Abstract

**Background:**

Hydatid disease or cystic echinococcosis (CE) is caused by the larval stages of the cestode parasite *Echinococcus granulosus*. The objectives of this study were to estimate the prevalence of seropositivity and to identify the risk factors associated with the disease among humans in Khartoum State, Central Sudan.

**Methods:**

A cross-sectional study was conducted between November 2017 and April 2018. A total of 305 randomly selected consenting participants from three localities were included in the current investigation using a multistage probability sampling method. An in-house enzyme-linked immunosorbent assay was used to detect immunoglobulin G antibodies to *E. granulosus*. The χ^2^ test and logistic regression analysis were used to determine the risk factors associated with CE seropositivity.

**Results:**

A seroprevalence of 6.5% (20/305) was recorded among humans in Khartoum State, Central Sudan. Age (odds ratio [OR] 16.61 [confidence interval {CI} 2.21 to 117.92], p=0.006), locality (OR 3.08 [CI 1.42 to 22.54], p=0.011) and contact with dogs (OR 2.34 [CI 0.026 to 0.646], p=0.013) were recorded as potential risk factors for seropositivity to CE in the study area.

**Conclusions:**

The seroprevalence of CE (6.5%) is high among humans in Khartoum State, Central Sudan. Improved surveillance is necessary to optimize control and prevention strategies for CE as an important neglected zoonotic disease among the human population in the study area of Central Sudan.

## Introduction

Cystic echinococcosis (CE), caused by the dog tapeworm *Echinococcus granulosus* (*EG*), accounts for 2–3 million cases of human echinococcal infections worldwide and constitutes a major public health problem in many parts of the world,^1–5^ including regions of South America, the Mediterranean countries, Eastern Europe, East Africa, the Near and Middle East, Central Asia and China.^11–15^

The life cycle of this cestode parasite requires a definitive carnivore host, usually dogs, and an intermediate host such as domestic or wild livestock. Equine and camelid animal species can also be infected with CE. Humans acquire the infection after accidental ingestion of eggs excreted with carnivore faeces and serve as a dead-end host of the parasite cycle.^9^

The disease is recognized as a neglected zoonotic disease by the World Health Organization.^6,7^ The economic impact of the disease may significantly affect the overall development and work productivity of humans in the endemic areas. The disease mainly affects pastoral communities in resource-poor settings. Unlike agricultural communities, pastoral communities usually have a higher prevalence of the disease due to the presence of a large population of dogs in the environment.^7–10^

Across Africa, CE is an endemic zoonotic disease in many parts of the continent. This includes Sudan; although the disease is of public health importance, it has been largely neglected in the past. However, a recent increase in interest by the Sudanese health authorities has led to several studies investigating the epidemiology of CE and the risk factors associated with the disease in humans. In southern Sudan, the overall prevalence was shown to be 3.5%, with 60% of the cysts located in the liver.^8^ In a survey conducted in humans in central Sudan, a cyst prevalence of 0.8% was reported using ultrasound.^9^ The prevalence of human hydatidosis was reported to be 1.22% in South Darfur State, with the predominant site of infection being the liver.^10^ Currently, 10 distinct genotypes of *EG*, designated as G1–G10, have been described worldwide. Of the 10 genotypes of *EG*, sheep (G1), cattle (G5) and camel (G6) strains have been reported among humans and livestock in Sudan.^2,16^ It is worth mentioning that the epidemiological studies on CE in Sudan were almost exclusively conducted in animals based on abattoir records.^9,17–19^ However, no information is available with regard to the prevalence and associated risk factors from a well-structured community-based study. Such epidemiological studies on CE in humans are essential to inform and appropriately implement control programs. Therefore, the present investigation was conducted among residents of Khartoum State in Central Sudan to determine the prevalence of CE as determined by detection of anti-*EG*-specific immunoglobulin G (IgG) antibodies and to examine variables considered as risk factors for acquiring the disease by applying a standardized questionnaire.

## Materials and methods

### Study area

Khartoum State is one of the largest states in Sudan, which includes three major cities: Khartoum, Bahry (Khartoum North) and Omdurman. Khartoum is the capital of Sudan and is located in the centre of the country. The population of Khartoum State is comprised of people from different parts of Sudan and is estimated to be nearly 6 million. The state covers an area of approximately 23 000 km^2^ and is situated at longitudes 31.5–34°E and latitudes 15–16°N, at the junction of the White Nile and the Blue Nile, forming the Nile River, which runs to the north throughout Sudan and Egypt. The climate is very hot and dry in the summer season, but cold and dry in the winter season. Average rainfall reaches 150 mm in the northeastern areas and 250 mm in the northwestern areas. The temperature in summer may reach up to 48°C from April to June. In winter, the temperature eventually declines to 15°C between November and January.

### Study design

A cross-sectional study was conducted from November 2017 to April 2018 within the three major cities of Khartoum State. Stratified sampling depended on the number of rural villages in each of the five municipalities. During field visits, epidemiological data were collected by a questionnaire that was written and administrated so all participants are asked precisely the same questions in an identical format. Responses were recorded in a uniform manner so as to increase its reliability. Blood samples were obtained for separation of sera and subsequently used in indirect enzyme-linked immunosorbent assays (ELISAs) for detection of seropositivity to anti-*EG*-specific IgG antibodies. A multistage probability sampling method was applied in this study. In the present study, a total of 305 randomly selected participants were included in the multistage probability sampling. The three localities (Khartoum, Omdurman and Khartoum North) were selected randomly from the five localities of Khartoum State. Ten villages were randomly selected in each locality and 10 houses were selected in each village. An additional five samples were taken, for a total of 305 participants.^20^

### Sample size

To estimate sample sizes, we used an echinococcosis prevalence of 3.5% in humans.^8^ A design effect of 2 and a non-response rate of 10% were used to adjust for the sampling technique. The formula for the calculation of the sample size was estimated using Epi Info 6.0 (www.cdc.gov/epiinfo/).^21^ The required sample size at 95% confidence, 3.5% prevalence and 0.025 absolute precision was calculated to be 209. This number was approximated to 305 samples to increase the accuracy of data obtained, of which 27, 157 and 121 were assigned to Bahry, Omdurman and Khartoum localities, respectively.

### Ethics approval and consent to participate

The study was approved by the Institutional Review Board of Al-Neelain University, Khartoum, Sudan. Information regarding the study was initially communicated to potential human participants prior to their signing an informed consent. The structured questionnaire was employed to collect risk factor–associated information.

### Questionnaire

The study included a questionnaire survey to determine the potential risk factors for transmission of CE in humans.^22^ The questionnaire included basic demographic data of the participants, data on education and occupation, living standards including waste management and water supply as well as slaughtering practices and knowledge of the disease (using visual material). Consented persons were interviewed and their responses transcribed to the questionnaire. Furthermore, the questionnaire was presented as open-format questions to reduce bias. Interviews were conducted for all participants in all above localities. Interviewers were trained before conducting the survey to ensure that the questionnaires were well understood by the participants so as to avoid differences in the definitions and interpretations of concepts used. All participants included in this study responded to the questionnaire, which covers sociodemographic characteristics including age (young age, <18 y; old age ≥18 y), gender (male and female), disease awareness (yes, no), occupation (employed, unemployed), education (illiterate, primary, secondary, university), locality, dog contact, occupation, presence of a dog in the house, dog treatment and home slaughtering.

### Antigen preparation

Scolices obtained from hydatids and hydatid cysts (the cyst originating from the lung after surgical removal) were washed five times in normal saline (0.9% sodium chloride). Soluble scolex antigen (SSA) was prepared after the disruption of whole scolices by frequent freezing and thawing. Scolices were further ultrasonically disrupted in a microcentrifuge ultrasonic disintegrator (Soniprep 150, MSE, Heathfield, UK).

The suspension was centrifuged at 12 000 rpm and the supernatant was collected. The deposit was suspended in phosphate-buffered saline (PBS, pH 7.2) and ultrasonically disintegrated. The supernatant was collected and added to the earlier supernatant. The combined supernatant was used as SSA antigen.^23^ The protein concentration of the antigen was estimated by the Biuret method using a spectrophotometer (UV-Vis Spectrophotometer UV/mini 1240, Shimadzu, Kyoto, Japan).^38^

### ELISA

Indirect ELISA was performed to screen the sera for *EG*-specific IgG antibodies as described previously, with some modifications.^23^ The ELISA was performed in 96-well immunoassay microplates (Nunc, Roskilde, Denmark) and optimal working dilutions of reagents were determined by chessboard titration.

Unless stated otherwise, 100 μl test volumes were used and incubations were performed for 1 h at 37°C. The plates were washed three times with PBS containing 1% Tween 20 (PBST; Merck, Darmstadt, Germany), wells were post-coated with 200 μl of PBS containing 2% bovine serum albumin (Calbiochem, La Jolla, CA, USA) and the diluents for reagents was PBS containing 10% skimmed milk (Khartoum, Vetrinary Molecular laboratory, Amba, Denmark). Briefly, the wells of polystyrene microtitration plates were coated with 100 µl of SSA, which was diluted in 0.05 M carbonate buffer of pH 8 to give a final concentration of 30 µg of protein/ml. The plates were then incubated overnight at 4°C. The plates were washed, and aliquots of test sera–positive and negative controls were added in separate wells at a dilution of 1:100. After another 1 h of incubation, the plates were washed and rabbit anti-human IgG conjugated horseradish peroxidase (HRP) was added to the plate at a dilution of 1:1000 and incubated again for 1 h. The plates were then washed and the substrate, 2,2-azino-bis(3-ethylbenthiazoline-6-sulfonic acid (Kirkegaard & Perry Laboratories, Gaithersburg, MD, USA) was added. Crimean–Congo haemorrhagic fever virus–infected camel serum was incorporated in each ELISA plate as a positive control to estimate the upper limit of the sensitivity. The results were read using an ELISA reader set at 405 nm. A presumptive diagnosis was made when the IgG antibody titre in the test sample had an optical density of <0.20. Positive control sera were obtained from human cases who were subjected to surgery for hydatid cyst removal. Sera from healthy uninfected individuals and patients infected with related cestodes *Taenia saginata* and *Taenia hydatigina* were used as negative controls.

### Statistical analyses

SPSS for Windows (version 21.0; IBM, Armonk, NY, USA) was employed to enter and analyse data. Univariable analysis using a χ^2^ test was used to determine the associations between the outcome variable (status of CE seropositivity) and its potential risk factors. A significant association between CE and a risk factor was initially considered as p<0.25 (two-tailed; α=0.25). The results of the univariable analysis were further subjected to multivariable analysis using logistic regression. The results were expressed as the odds ratio (OR) with 95% confidence interval (CI) for each risk factor. A p-value <0.05 was interpreted as representing a significant association between CE seropositivity and the associated risk factor.

## Results

The initial ELISA screening for anti-*EG*-specific IgG antibody, revealed positive results in 20 of 305 serum samples, which accounts for a seroprevalence of 6.5%. The highest rate of CE seropositivity was recorded in Khartoum (14.8%), whereas the lowest rate of seropositivity was recorded in Omdurman (0.6%).

The univariate analysis using a χ^2^ test was conducted for the association between the potential risk factors and CE seropositivity. The initial results using univariate analysis showed that the independent variables were statistically significant; including age, locality, dog contact, occupation, presence of a dog in the house, dog treatment and home slaughtering (p<0.25; Table 1). The significant results in the univariable model were further subjected to a final multivariate model using logistic regression analysis to illuminate confounding factors. Age (OR 16.61 [CI 2.21 to 117.92], p=0.006), localities (OR 3.08 [CI 1.42 to 22.54], p=0.011) and contact with dogs (OR 2.34 [CI 0.026 to 0.646], p=0.013) were recorded as potential risk factors for seropositivity to CE. The results are summarized in Table 2. Other potential factors did not show any significant association with CE seropositivity.

**Table 1.** Summary of analysis for risk factors of echinococcosis (IgM) among humans in Khartoum State, Sudan (n=305) using a χ^2^ test

Risk factors	Animals tested, n	Animals affected, n (%)	fd	χ^2^	p-Value
Locality			2	20.09	0.001
Bahry	27	2 (7.4)			
Omdurman	157	1 (0.6)			
Khartoum	121	17 (14.8)			
Age			1	12.48	0. 001
Young	162	3 (1.9)			
Old	143	17 (11.9)			
Gender			1	0.027	0. 86
Female	193	13 (6.7)			
Male	112	7 (6.2)			
Education			3	0.74	0.87
Illiterate	55	5 (9.1)			
Primary	72	4 (5.6)			
Secondary	62	4 (6.5)			
University	116	7(6.0%)			
Dog contact			1	5.18	0.136
No	186	17 (9.1)			
Yes	119	3 (2.5)			
Occupation			4	6.90	0.141
Teacher	21	2 (9.5)			
Housewife	109	6 (5.5)			
Retiree	20	4 (20)			
Employer	94	5 (5.3)			
Student	61	3 (4.9)			
Presence of dog			1	1.86	0.145
No	257	19 (7.4)			
Yes	48	1 (2.1)			
Dog treatment			1	10.2	0.001
No	206	20 (9.7)			
Yes	99	0 (0.0)			
Home slaughterhouse			1	1.50	0.220
No	24	3 (12.5)			
Yes	281	17 (6.0)			

**Table 2.** Multivariate analysis using a logistic regression model for significant association (p<0.05) of 426 risk factors and seropositivity (IgG) among humans in Khartoum State

Risk factors	OR	95% CI	p-Value	
Age				
Young	Ref.	2.21 to 117.92	0.006	
Old	16.61			
Locality				
Omdurman	Ref.	1.42 to 22.54	0.011	
Khartoum	3.08			
Dog contact				
No	Ref.	0.026 to 0.646	0.013	
Yes	2.34			

## Discussion

CE in humans and animals is characterized by the development of metacestode larval stages in the liver and other organs. CE is a relevant public health and economic problem worldwide.^1,2,24–26^ The disease is endemic in many areas of the African continent, including Sudan.^17–19,27–33^ However, due to the lack of epidemiological data, problems associated with disease diagnosis and the chronic nature of infection and long-term treatment, it often has a low priority and is therefore part of the group of neglected tropical diseases.^7^

Several immunodiagnostic assays have been employed in serological studies to assess the prevalence and associated risk factors of CE in humans worldwide.^13,21,34^ Most of the described serological assays did not detect all cases of CE in infected humans. This is mainly attributed to the low sensitivity and specificity of the serological assays compared with field studies, which were conducted using imaging techniques such as portable ultrasound.^35,36^

The present study showed that the prevalence of African horse sickness virus (AHSV) IgG-specific antibodies was high (5.6%). IgG-specific antibodies against *EG* recorded in this study showed evidence of prior exposure of humans to CE in Khartoum State. The high prevalence rate (6.5%) indicates significant circulation of the disease among horses in Central Sudan. Seven parameters considered as risk factors with p<0.25 (two-tailed; α=0.25) were initially considered to be associated with seropositivity to CE among humans in Khartoum State in the univariate analysis using the χ^2^ test. These included dog contact, age, dog treatment, presence of a dog in the house, localities, occupation and home slaughtering. However, the multivariate analysis using a logistic regression model illustrated that only three potential risk factors were significantly associated with CE seropositivity in the study area, including dog contact (OR 2.34 [CI 0.026 to 0.646], p=0.013).

The study showed that humans in contact with dogs are at least twice as likely to be at risk of acquiring an echinococcal infection. This is attributed to the fact that the patients are likely to become infected through the contaminated environment with infected dog faeces that contains *EG* eggs. The highest rate of CE seropositivity was recorded among humans in Khartoum (14.8%). This is most probably due to the presence of a high-density dog population in this locality, which provides parasite eggs for human infection with CE. There was also an association between CE seropositivity and Khartoum (OR 3.08 [CI 1.42 to 22.54], p=0.011), again due to high population density of dogs in this locality. Residents of Khartoum are at 3-fold risk of becoming infected with CE compared with other localities, suggesting an increased endemicity of this locality with echinococcosis infection. Age was also reported as a potential risk factor in this study (OR 16.61 [CI 2.21 to 117.92], p=0.006). Of interest, participants >18 y of age are at a very high risk, as they are 16 times more likely to become infected with CE. The present study illustrated that the prevalence of CE seropositivity is alarmingly high among the population of Khartoum State and is probably similarly high throughout the country.

It appears that the survey should be extended in the future to include prevalence and associated risk factors of echinococcal infection among dog populations. Molecular epidemiological studies should also be considered to identify the genotypes of the parasites circulating in the country. No significant difference was reported between CE seropositivity and other potential risk factors included in the study. Males and females were equally affected by CE and hence gender had no significant association with CE seropositivity. In addition, no significant difference was observed between AHSV seropositivity and age or education status. Early diagnosis of CE is necessary for prevention and control of the disease.

It should be noted that long follow-up treatment with albendazole is recommended for treatment of CE in infected patients. Dog owners should also be educated an the risks of the disease and prevention of the infection through proper hygienic measures such the management of waste, waste handling, washing hands and the use of plastic gloves when cleaning a dog facility. Dewormer for dogs should be used at regular intervals to ensure the elimination of the adult *EG*. In this regard, effective animal husbandry and management systems should be applied to control dog breeding.

In an endemic area such as Khartoum State, where the main factors for seropositivity are those linked to contact with dogs, it is extremely important to focus on interruption of the life cycle of the parasite to prevent the spread of infection from dog to human. However, it should be noted that in stray dogs, this method of control is extremely difficult, if not impossible. Therefore, an important intervention would be to increase the frequency of application of anthelmintic drugs. Control measures should be directed towards education and animal management to disrupt the life cycle of the parasite.^37^

Currently the Sudan Ministry of Health has taken these recommendations into account for a proper control program to combat this important zoonotic infection. The epidemiology of human CE is complex and depends on the presence of the parasite in the zoonotic cycle, which involves a larval stage host such as livestock, equines and wildlife. This study suggested that hydatid disease is maintained in the study area mainly by the existence of large numbers of dogs, which shed infective eggs into the environment.^38^ Improper hygiene measures and the presence of stray dogs in the country provide ideal conditions for maintenance of the life cycle of this parasite, as they have an increased risk of acquiring *EG* infection by having free access to infected carcasses.^39–41^ In this study, we estimated the prevalence of human echinococcosis and the associated risk factors in Khartoum State. We anticipate that this study will help to facilitate control programs aimed at controlling the infection in dogs and preventing new infections in humans.

### Limitations of the study

SSA was employed in our ELISA. A more defined recombinant antigen would be recommended to improve the specificity of the ELISA by eliminating the possibility of cross-reaction with other related cestodes. Further studies will be required to verify this finding, such as prospective studies with a larger sample size. In addition, the questionnaire did not include information related to infection with *EG* in dogs. It is therefore recommended that further studies on canine echinococcosis should be conducted in parallel with human CE to better predict and respond to the disease in the study area of Central Sudan.

## Conclusions

The results obtained from the present study confirmed the circulation of CE in Khartoum State as determined by indirect ELISA. The seroprevalence of CE is high (6.5%) among residents of this state. Age, locality and contact with dogs were identified as potential risk factors for contracting the disease. The genotypes of the hydatid cysts in infected patients in the area remain to be identified. Surveillance for CE among residents of the state and the distribution among the dog population should continue for a better understanding of the epidemiology of the disease. This study provides suggestions to the public health authorities regarding control of the disease and prevention of spread of the infection to the human population in Khartoum State.
